# Relapsing polychondritis with large airway involvement

**DOI:** 10.1002/rcr2.501

**Published:** 2019-11-12

**Authors:** Christiaan Yu, Simon A. Joosten

**Affiliations:** ^1^ Monash Lung and Sleep Monash Health Clayton Victoria Australia

**Keywords:** Non‐invasive ventilation, relapsing polychondritis, tracheomalacia

## Abstract

Relapsing polychondritis is a rare autoimmune condition characterized by episodic and progressive cartilaginous inflammation. Its clinical presentation is vastly divergent and can affect various organs. We report the uncommon case of large airway involvement in a patient presenting with shortness of breath on the background of diagnosed relapsing polychondritis. Computed tomography (CT) chest demonstrated thickening of the cartilaginous portions of the trachea and bronchi with sparing of the posterior membranes, consistent with tracheobronchomalacia and repeated cartilaginous destruction. High doses of systemic glucocorticoids, accompanied by continuous positive airway pressure, were required for treatment. We highlight the importance of identifying the extent of airways affected and definitive positive airway pressure support for relapsing polychondritis affecting major airways in addition to conventional therapy of immunosuppression.

## Introduction

Relapsing polychondritis is a rare life‐threatening autoimmune condition. The exact incidence of this disease is unknown. Clinical presentation varies from auricular chondritis and polyarthritis to large airway involvement. The disease can be stabilized on immunosuppression; however, exacerbations may be life‐threatening depending on the organs involved. We present a previously undescribed occurrence of relapsing polychondritis with acute large airway involvement requiring short‐term positive airway pressure therapy in the context of a respiratory viral infection.

## Case Report

A 57‐year‐old woman with a past history of relapsing polychondritis presented with acute shortness of breath and wheeze after a long‐distance flight.

In her 30s, she was diagnosed with relapsing polychondritis based on McAdam's criteria (bilateral auricular chondritis, nasal chondritis, ocular inflammation, and respiratory tract chondritis) 1. Her condition had been stable on 5 mg of prednisolone daily and 20 mg of methotrexate weekly for the past few years. Previous attempts to wean off immunosuppression led to exacerbations requiring high doses of prednisolone. Her baseline exercise tolerance was good, and she could walk up to 2 km. Her MRC dyspnoea scale was grade 1.

**Figure 1:**
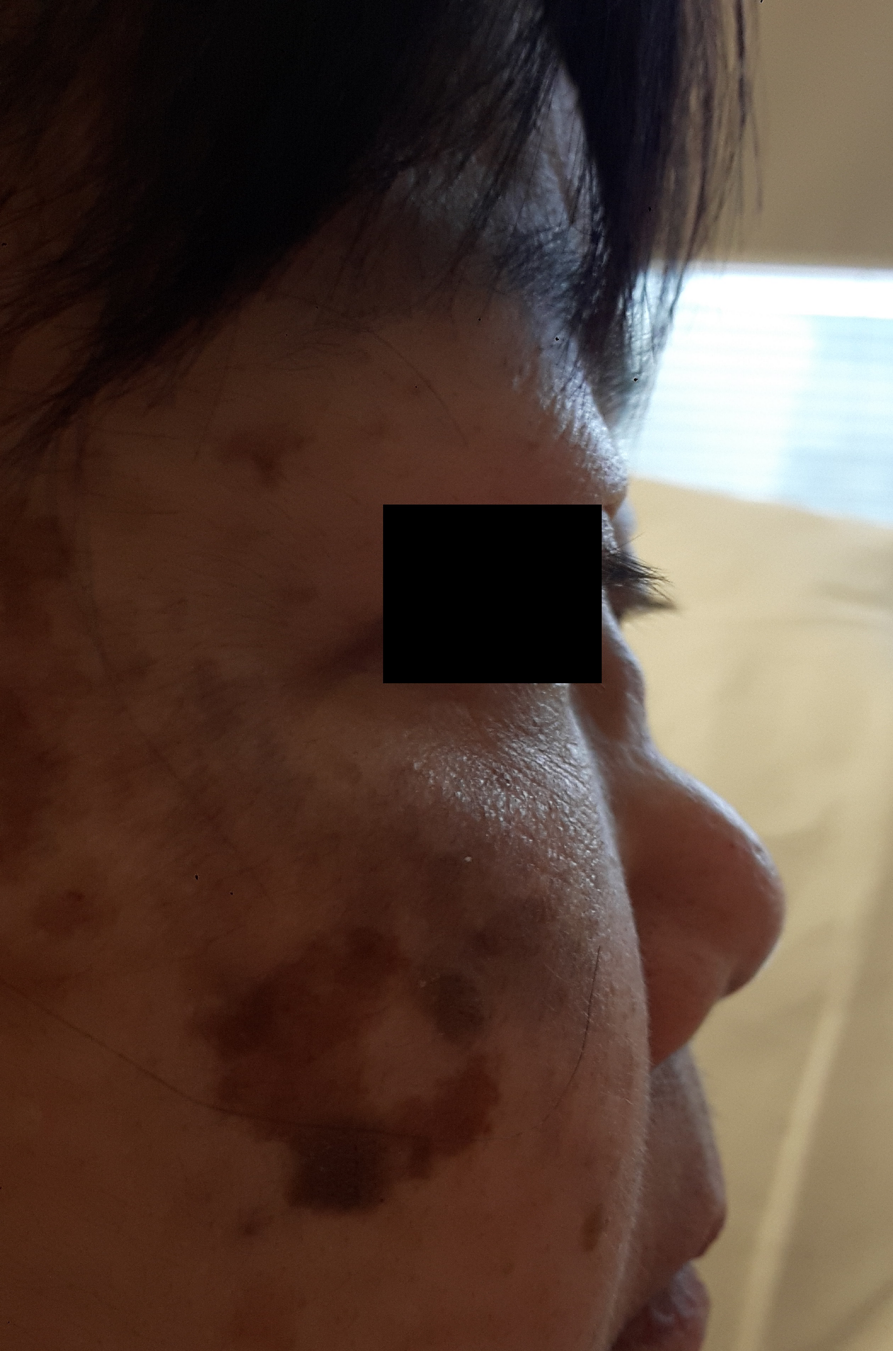
Saddle nose deformity resulting from nasal chondritis.

**Figure 2:**
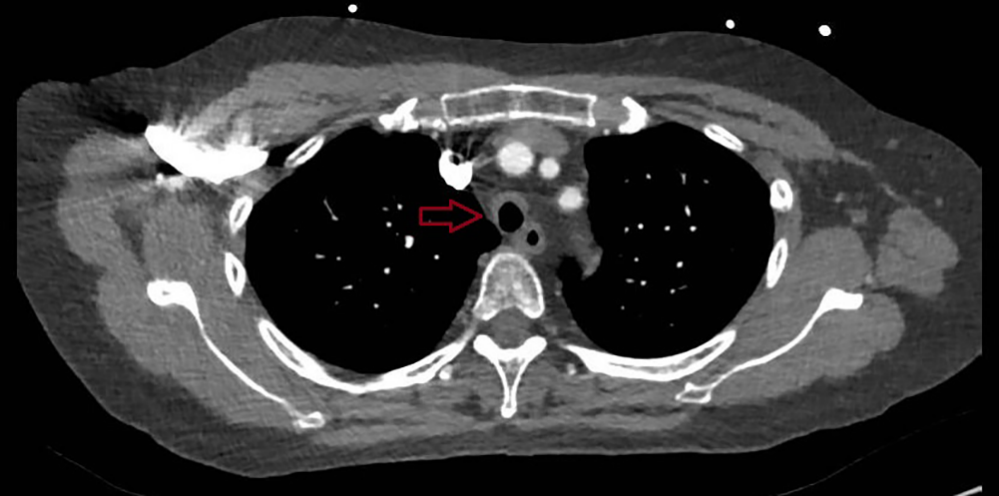
Computed tomography (CT) chest showing smooth thickening of cartilaginous portions of the trachea (anterior and lateral walls) with sparing of the posterior membranes.

On initial examination, her heart rate was 130 beats per minute (regular), blood pressure 160/90, respiratory rate 24 breaths per minute, and oxygen saturation 94% on room air. Clinical examination demonstrated a prominent saddle nose deformity (Fig. 1), loss of cartilage integrity in both ears, and expiratory wheeze throughout both lung fields and over the larynx. CT pulmonary angiogram showed thickening of the cartilaginous portions of the trachea and bronchi with sparing of the posterior membranes (Fig. 2), consistent with tracheobronchomalacia secondary to repeated cartilaginous inflammation, with alternative differential diagnoses including viral tracheobronchitis. Of note, there was no pulmonary embolus detected. Nasopharyngeal aspirate was positive for parainfluenza virus 3 RNA. There was no evidence of a concomitant vasculitis as her antinuclear antibodies (ANA) and antineutrophil cytoplasmic antibodies (ANCA) were both negative. Kidney function was within normal limits, estimated glomerular filtration rate (eGFR) >90, Cr50. Erythrocyte sedimentation rate (ESR) was not elevated at 13.

In the first 24 h, she was treated with 50 mg of prednisolone and regular bronchodilators (salbutamol and ipratropium) but remained short of breath and subsequently desaturated to an oxygen saturation of 89%. Repeated venous blood gases indicated a normal pH and PaCO_2_ within normal limits. Upon commencement of continuous positive airway pressure up to 8 mmH_2_O, the patient's condition improved significantly. She required supplemental oxygen up to FiO_2_ 0.26% for her hypoxia, which resolved following positive airways pressure therapy. She was on continuous positive airway pressure therapy for 24 h a day for 2 days followed by overnight for 3 days, which was then was completed ceased. Pressures were titrated to symptoms and tolerability. Based on her clinical response to treatment, dynamic CT imaging and bronchoscopy was not performed. During her admission, there were no extra pulmonary clinical manifestations of relapsing polychondritis. After a week of treatment, she was discharged home with a prolonged wean of corticosteroids.

Six months following this episode, the patient has progressed well and is back on her initial immunosuppression regime with no further exacerbations.

## Discussion

Relapsing polychondritis is a rare autoimmune condition characterized by episodic and progressive cartilaginous inflammation 2. It may affect virtually any organ in the body, typically cartilages of the nose, ears, large airways, and ribs but also large vessels, kidneys, skin, and the heart. The clinical course follows a relapsing remitting pattern with flares varying in severity. The aetiology of this condition is unknown, and pathogenesis involves autoimmunity towards connective tissue epitopes following an initial insult 3. The exact incidence of this condition is unclear, and there are only a few published single‐centre case series from across the world, with one of the largest series quoting 158 cases over 28 years 4.

Poor prognostic factors have previously been recognized and include the presence of a saddle nose deformity, anaemia and systemic vasculitis 5.

Large airway involvement is common and is a major cause of mortality and morbidity, present in up to 50% of patients 6. In the acute setting, it is critical to diagnose and treat large airway involvement to institute early management to avoid an emergency tracheostomy. Definitive positive airway pressure therapy prevents the dynamic collapse during inspiration and expiration caused by the loss of structural cartilaginous support. It is vital to identify laryngotracheal disease promptly as recurrent episodes of inflammation and destruction lead to life‐threatening tracheobronchomalacia and critical airway stenosis. This may require further airway interventional therapy such as stent placement, tracheostomy, long‐term continuous positive airway support, or surgery.^7^, ^8^

Systemic glucocorticoids are the mainstay of treatment to dampen the immune response in moderate to severe disease. Other forms of immunosuppression may be indicated depending on the response to prednisolone and other organs involved 9.

We highlight the clinical challenge of the management for this condition and raise awareness of tracheobronchial involvement in relapsing polychondritis. Positive airway pressure therapy may be used in the acute setting for exacerbations that are self‐limiting without the need for longer‐term therapy. This would provide immediate airway support, augmenting the slower‐acting immunosuppression.

### Disclosure Statement

Appropriate written informed consent was obtained for publication of this case report and accompanying images.

## References

[1] McAdam LP , O'Hanlan MA , Bluestone R , et al. 1976 Relapsing polychondritis: prospective study of 23 patients and a review of the literature. Medicine (Baltimore) 55:193–215. 775252

[2] Trentham DE , and Le CH . 1998 Relapsing polychondritis. Ann. Intern. Med. 129:114–122. 9669970 10.7326/0003-4819-129-2-199807150-00011

[3] Foidart JM , Abe S , Martin GR , et al. 1978 Antibodies to type II collagen in relapsing polychondritis. N. Engl. J. Med. 299:1203–1207. 714080 10.1056/NEJM197811302992202

[4] Lin DF , Yang WQ , Zhang PP , et al. 2016 Clinical and prognostic characteristics of 158 cases of relapsing polychondritis in China and review of the literature. Rheumatol. Int. 36:1003–1009. 26951051 10.1007/s00296-016-3449-8

[5] Michet CJ Jr , CH MK , Luthra HS , et al. 1986 Relapsing polychondritis. Survival and predictive role of early disease manifestations. Ann. Intern. Med. 104:74–78. 3484422 10.7326/0003-4819-104-1-74

[6] Sharma A , Gnanapandithan K , Sharma K , et al. 2013 Relapsing polychondritis: a review. Clin. Rheumatol. 32:1575–1583. 23887438 10.1007/s10067-013-2328-x

[7] Cansiz H , Yilmaz S , and Duman C . 2007 Relapsing polychondritis: a case with subglottic stenosis and laryngotracheal reconstruction. J. Otolaryngol. 36:E82–E84. 18076833

[8] Zhang JQ , Li Q , Bai C , et al. 2007 Clinical features and treatment of relapsing polychondritis with involvement of the respiratory tract‐report of thirteen cases. Zhonghua Jie He He Hu Xi Za Zhi 30:173–177. 17572994

[9] Vitale A , Sota J , Rigante D , et al. 2016 Relapsing polychondritis: an update on pathogenesis, clinical features, diagnostic tools, and therapeutic perspectives. Curr. Rheumatol. Rep. 18:3. 26711694 10.1007/s11926-015-0549-5

